# Feasibility of a Real-Time Clinical Augmented Reality and Artificial Intelligence Framework for Pain Detection and Localization From the Brain

**DOI:** 10.2196/13594

**Published:** 2019-06-28

**Authors:** Xiao-Su Hu, Thiago D Nascimento, Mary C Bender, Theodore Hall, Sean Petty, Stephanie O’Malley, Roger P Ellwood, Niko Kaciroti, Eric Maslowski, Alexandre F DaSilva

**Affiliations:** 1 Headache & Orofacial Pain Effort Lab, Biologic & Materials Sciences Department School of Dentistry University of Michigan Ann Arbor, MI United States; 2 Center for Human Growth and Development University of Michigan Ann Arbor, MI United States; 3 3D Lab, Digital Media Commons University of Michigan Ann Arbor, MI United States; 4 Clinical Method Development Colgate Palmolive Piscataway, NJ United States; 5 Department of Biostatistics, School of Public Health University of Michigan Ann Arbor, MI United States; 6 Moxytech Inc Ann Arbor, MI United States

**Keywords:** pain, spectroscopy, near-infrared, virtual reality, artificial intelligence

## Abstract

**Background:**

For many years, clinicians have been seeking for objective pain assessment solutions via neuroimaging techniques, focusing on the brain to detect human pain. Unfortunately, most of those techniques are not applicable in the clinical environment or lack accuracy.

**Objective:**

This study aimed to test the feasibility of a mobile neuroimaging-based clinical augmented reality (AR) and artificial intelligence (AI) framework, CLARAi, for objective pain detection and also localization direct from the patient’s brain in real time.

**Methods:**

Clinical dental pain was triggered in 21 patients by hypersensitive tooth stimulation with 20 consecutive descending cold stimulations (32°C-0°C). We used a portable optical neuroimaging technology, functional near-infrared spectroscopy, to gauge their cortical activity during evoked acute clinical pain. The data were decoded using a neural network (NN)–based AI algorithm to classify hemodynamic response data into pain and no-pain brain states in real time. We tested the performance of several networks (NN with 7 layers, 6 layers, 5 layers, 3 layers, recurrent NN, and long short-term memory network) upon reorganized data features on pain diction and localization in a simulated real-time environment. In addition, we also tested the feasibility of transmitting the neuroimaging data to an AR device, HoloLens, in the same simulated environment, allowing visualization of the ongoing cortical activity on a 3-dimensional brain template virtually plotted on the patients’ head during clinical consult.

**Results:**

The artificial neutral network (3-layer NN) achieved an optimal classification accuracy at 80.37% (126,000/156,680) for pain and no pain discrimination, with positive likelihood ratio (PLR) at 2.35. We further explored a 3-class localization task of left/right side pain and no-pain states, and convolutional NN-6 (6-layer NN) achieved highest classification accuracy at 74.23% (1040/1401) with PLR at 2.02.

**Conclusions:**

Additional studies are needed to optimize and validate our prototype CLARAi framework for other pains and neurologic disorders. However, we presented an innovative and feasible neuroimaging-based AR/AI concept that can potentially transform the human brain into an objective target to visualize and precisely measure and localize pain in real time where it is most needed: in the doctor’s office.

**International Registered Report Identifier (IRRID):**

RR1-10.2196/13594

## Introduction

### Background

Accurate pain assessment is crucial across a wide range of acute and chronic pain conditions to provide proper diagnosis and treatment, especially when patients have limitations to express their ongoing suffering. The estimated economic impact of pain, from direct medical costs to loss of productive time, is US $560 to $635 billion every year [1]. Despite this, we are still heavily relying on the following measure: “From 0 to 10—0 being no pain and 10 being the worst pain—what is your pain level?” All of our clinical and research decisions on the efficacy of current or new potential pain therapies and, most importantly, our patients’ ongoing pain levels, are biased by the inaccuracy of that scale, independent of how rigorous, complex, and costly our protocols are.

The pain field has progressed by quantifying the patients’ suffering with more holistic pain questionnaires and measure scales (eg, McGill Pain Questionnaire and Face Rating Pain Scale), which are prevalent, useful, and convenient. However, the subjective reports still carry limitations: first, they are inconsistent among different patient groups regarding age and cultures. For instance, words used by patients nowadays to express the severity of their pain have also evolved with time and might be different from the ones articulated by past generations [2]. Second, those tools cannot be applied during procedures or surgeries that impair patients’ communication, including the minimally conscious or cognitively impaired. Finally, self-report provides limited value for understanding the neurophysiological processes underlying different types of pain, thereby, blurring the treatments to the underlying neuropathologic conditions [3].

To address these limitations, researchers have started to analyze the neurological signature of pain using neuroimaging [3,4]. Wager and colleagues developed a system using machine learning technology on data collected with functional magnetic resonance imaging (fMRI), showing the possibility of detecting a robust neurological signature of pain at the level of the individual person. Other researchers have demonstrated the possibility of detecting even temporomandibular disorder using multivoxel pattern analysis on fMRI signal. Such MRI-based gold rush to report new brain-pain biomarkers forced the field to recommend standards of evidence [5]. Indeed, fMRI objective assessments of pain have provided a great step ahead in the path of dissecting brain mechanism of pain, but the size and cost of the MRI scanner and other conventional neuroimaging tools (eg, positron emission tomography) prevent its application in the clinical office. This impediment has sparked the interest of portable neuroimaging devices with similar technical benefits as fMRI. Functional near-infrared spectroscopy (fNIRS) detects concentration variations of oxygenated hemoglobin (HbO) and deoxygenated hemoglobin (HbR), such as blood oxygen level dependent signal in fMRI. It measures the absorption of near-infrared light at wavelengths between 700 and 1000 nm, noninvasively through the skull [6,7]. Compared with the MRI scanner, the portability and compatibility to ferromagnetic/electrical components provide researchers an option to monitor, localize, and analyze functional brain activity in the surgical and clinical environment [8-12].

### Objectives

In previous studies, our group studied the hemodynamic cortical responses detected by fNIRS in patients with hypersensitive teeth in the dental chair [13]. We found well-defined hemodynamic cortical activity in the primary sensory (S1) and prefrontal cortices (PFCs) elicited by thermal stimulation to the affected tooth from expectation to pain detection. Interestingly, the patients’ clinical pain experience was predicted concomitantly by their baseline functional connectivity between S1 and PFC, as well as a well-defined stepwise sequence of hemodynamic responses. This sensory-discriminative and cognitive-emotional cascade of brain responses initiated during the expectation of the clinical pain (prepain phase), with activations in the contralateral S1 orofacial homuncular region and also in the bilateral PFC. Such activations were followed by flat or PFC deactivation and further S1 responses when the cold stimuli crossed noxious levels (pain phase) [14]. Herein, following our earlier findings, we aimed to develop an in-house framework technology that can visualize, measure, and decode in real time the ongoing cascade of spread cortical activities into when and where there is clinical pain. This was successfully achieved through 3-step experiments integrating optical neuroimaging (fNIRS), augmented reality (AR), and a neural network (NN)–based artificial intelligence (AI).

## Methods

### Data Acquisition

The University of Michigan Institutional Review Board approval was obtained before study initiation. We recruited 21 participants (8 male; age: mean 27.6, SD 3.5 years) with hypersensitive teeth. We collected neuroimaging data from a thermal stimulation session [13,14]. In this session, the participants underwent 20 thermal stimulation trials, in which the thermal probe cycled from 32^o^C to 0^o^C at a rate of –2^o^/second. Subjects controlled the cooling unit by clicking a computer mouse when clinical pain was achieved, causing the stimulation to stop.

The data were acquired with a TechEN-CW6 fNIRS (Milford, MA, United States) system at a 20 Hz sampling rate. The setup included 8 emitters of near-infrared light and 28 detectors spaced 3 cm apart, yielding 40 data channels deployed at bilateral PFC and S1. The probe set was designed based on the international 10-10 transcranial positioning system [15] and further validated with automatic anatomical labeling database [16]. The collected raw data were examined by a 2-step data quality control steps, filtered with a band-pass filter at 0.01-0.3 Hz and then converted to HbO and HbR concentration change data. Such preprocessing was completed using scripts from Homer2 software (Huppert et al) [17] and several custom MATLAB (MathWorks, MA, United States) scripts.

**Figure 1:**
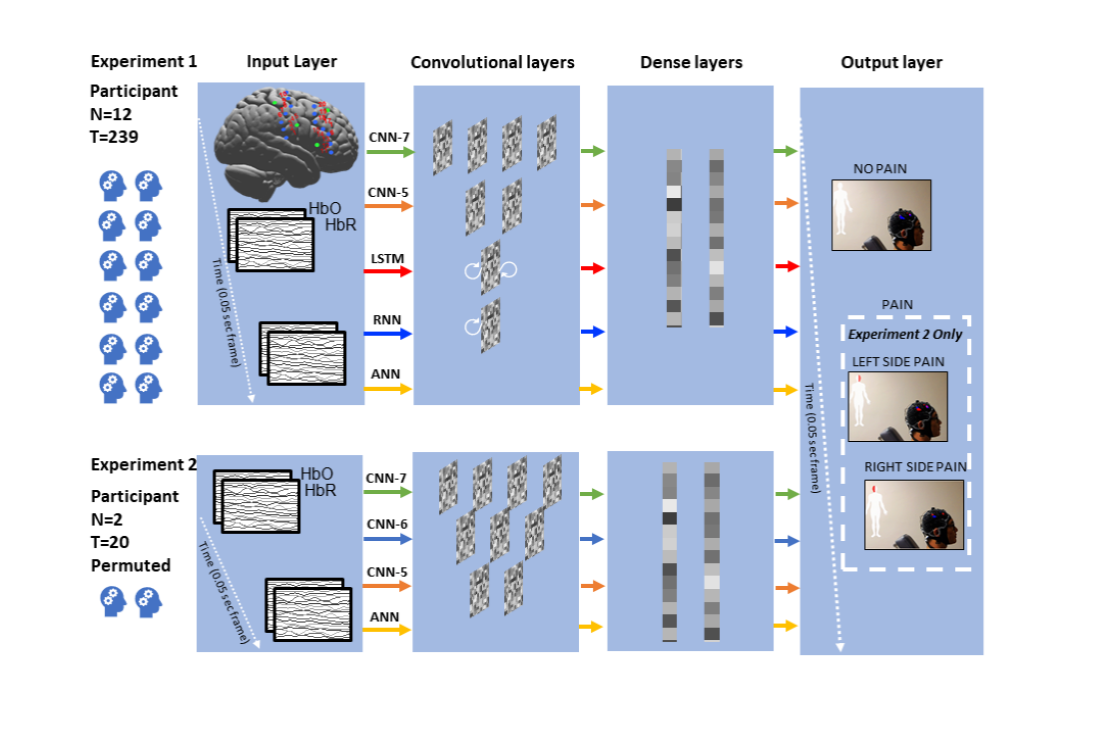
Experiment flow chart. The green line indicates the convolutional neural network with 7 layers (CNN-7), the blue line indicates the CNN network with 6 layers, the orange line indicates the CNN network with 5 layers (CNN-5), the red line indicates the long short-term memory network, the dark blue line indicates the recurrent NN, and the yellow line indicates the artificial NN with 3 layers for experiment 1—pain/no-pain prediction and experiment 2—left/right pain localization task. Experiment 1 included the data collected from N=12 participants, 239 trials in total, whereas experiment 2 included the data collected from N=2 participants, 20 trials in total. CNN: convolutional neural network.

**Figure 2:**
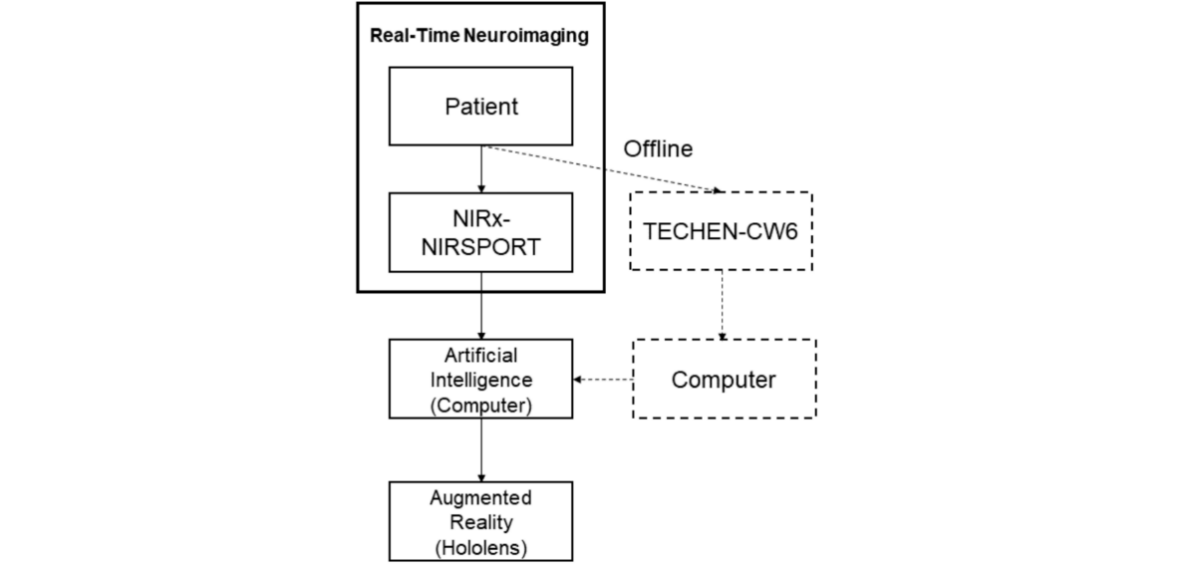
Study framework.

In this study, we employed 2 experiments for testing the feasibility of pain/no-pain prediction as well as left/right pain localization (Figure 1). In addition, we conducted a third experiment of designing an AR-based data visualization terminal. The entire study framework is presented in Figure 2.

The NN design, training, and testing were completed in a Python-based toolbox, Keras (Chollet et al) [18], with Tensorflow backend (Google Brain Team) [19] and Scikit-learn (Cournapeau et al) [20] toolbox for cross-validation. The data displaying terminal on the AR device were designed through HoloBrain (Microsoft, WA, United States), an in-house developed software at University of Michigan.

### Experiment 1: Pain Detection

The aim of the experiment was to test the feasibility of pain/no-pain prediction at individual patient level. We tested convolutional NN (CNN) configurations at 3 different depths, respectively, 7 layers (CNN-7), 5 layers (CNN-5), and 3 layers (artificial NN, ANN) to evaluate their performance on same datasets (Figure 1). In addition, we also tested a recurrent NN and a long short-term memory network on our dataset considering the possible temporal connection within fNIRS time series. Prior studies suggested that including data history as feature can improve classification performance [9,21]. In our most recent research, we reported the cascade of brain events during clinical pain that demonstrated interactive pain expectation evoked responses at bilateral prefrontal cortices as well as a 2-peak response at contralateral sensory cortex [13]. Using that knowledge in this study, we assembled 2 types of feature for the input layer: (1) a 40 × 40 × 2 data cube, by including 40 samples (2-second data history block sampled at 20 Hz) and 40 channels with 2 types of data (HbO/HbR) in the third dimension and (2) a 80 × 40 × 2 data cube, by including 80 samples (2 × 2-second data history blocks before and after patients’ pain threshold). Given that there were more no-pain than pain samples in the dataset within the multiple clinical stimulation trials in each individual, we balanced the 2 sample sets by reweighting their loss functions during the training process [22]. Specifically, we defined a dictionary for the pain/no-pain labels with associated weights of 10:1 and assigned such weight during training in the Keras toolbox using a class_weight variable. In addition, we used 10-fold cross-validation to validate each model [23] and calculated the averaged classification accuracy, sensitivity, specificity, positive likelihood ratio (PLR), positive predictive value, negative predictive value, and kappa value to evaluate the classification performance.

### Experiment 2: Pain Localization

The aim of the experiment was to further test the feasibility of left/right pain and no-pain states prediction (3-class classification) on merged and permuted patients’ data (data were collected from patient 3 and 19, separately, left/right tooth stimulated). We permuted the merged data by randomly including and excluding data cubes along time course. We tested all networks applied in experiment 1, and in addition a 6-layer CNN (CNN-6) on type I data cube, with time series preprocessed with a custom real-time normalization algorithm (Figure 1). Specifically, we used a divided by the mean [24] scheme to normalize the data in real time, where the mean was updated by a windowed data along the time course. To retain the uniformity in the data, we did not run cross-validation in this scenario.

### Experiment 3: Augmented Reality—Pain Visualization and Decoding Using Augmented Reality and Artificial Intelligence

We developed a displaying terminal for the framework using an AR device, HoloLens (Microsoft, WA, United States). AR is a computer vision–based technology that expands our real world by adding a layer of virtual and digital information to it. It is becoming prevalent in different fields including, for example, construction, gaming, and medicine. The Hololens is a headset-shaped AR computer developed by Microsoft, which allows users to visualize 3-dimensional (3D) holographic images on top of the real physical world. In this study, the functional hemodynamic response data acquired from the patient’s brain at multiple cortical regions of interest were wirelessly transmitted to the HoloLens device. Afterwards, we used an in-house–developed software to display the ongoing patient’s cortical function updating in real time on the brain template modeled in the software (Multimedia Appendix 1; video section 1 and 2). This software is an application adapted from an in-house 3D rendering engine developed at the University of Michigan 3D Lab. In active development for over 10 years, this platform allows for the rapid development and displaying of complex interactive 3D scenes including advanced materials, lighting, physical responses, and detailed meshes. In this study, we first registered the 40 data acquisition channels to an MNI 152 nonlinear brain template. Afterwards, we reconstructed this virtual brain with the registered functional regions using this software to display and adjust its appearance based on incoming data from the NIRS device. Furthermore, the software decoded and displayed the brain activity from experiments 1 and 2 in clinical pain/no-pain status and localization by mapping the ongoing results on a virtually reconstructed digital body within the field of AR view (Multimedia Appendix 1; video section 2). In addition, the CLARAi displaying of ongoing cortical activity in volunteers was also tested in real time using a NIRSport fNIRS system with 16 source-detector density (NIRx, NY, United States) to facilitate the use of our CLARAi concept in real clinical environment (Multimedia Appendix 1; video section 1).

## Results

### Experiment 1 and 2

**Figure 3:**
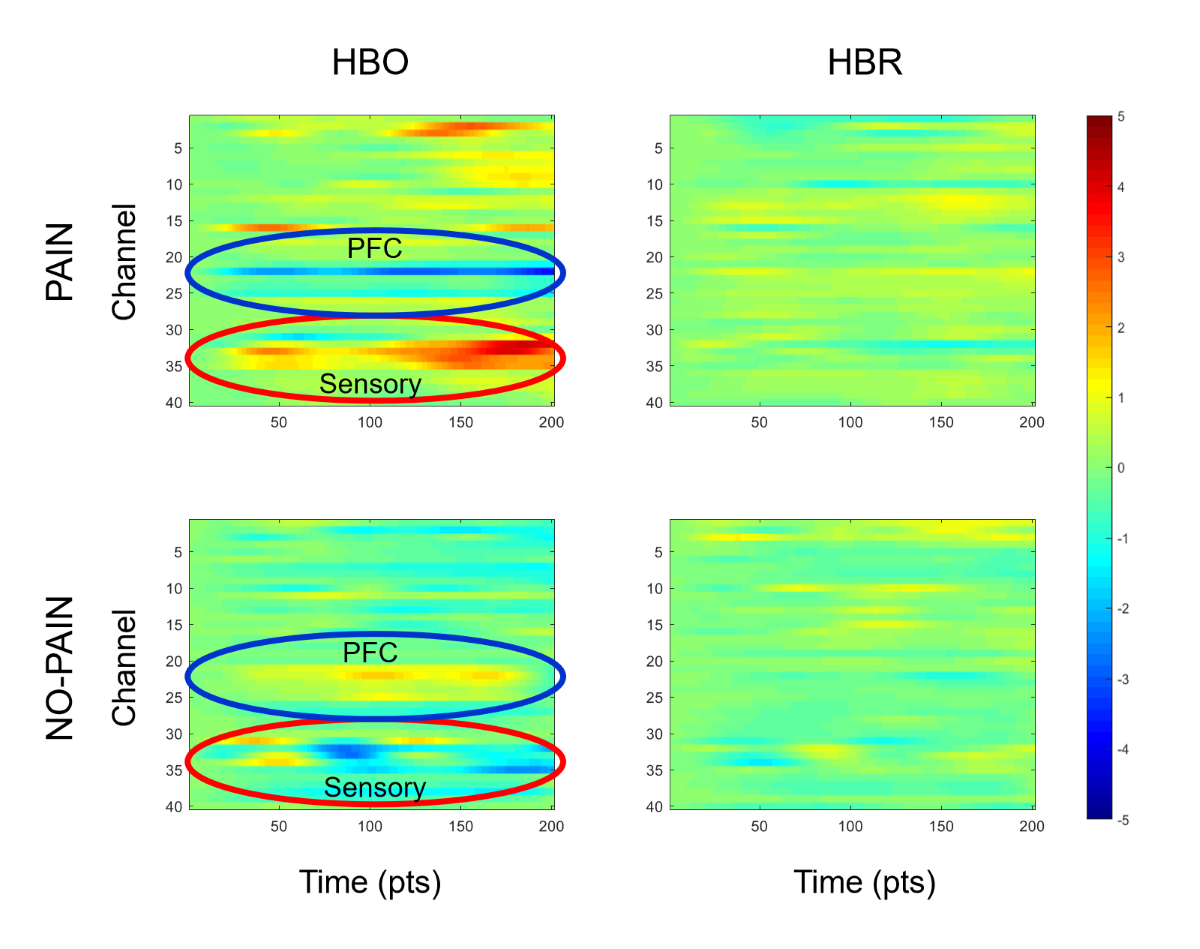
Representative averaged oxygenated hemoglobin (HbO) and deoxygenated hemoglobin (HbR) heat map from all data channels. The upper and lower panels, respectively, indicated the hemodynamic responses during pain and no-pain statues. The left and right panels, respectively, indicated the HbO and HbR responses. The red and blue circles, respectively, highlighted 2 regions of interest, sensory and prefrontal cortices. HbO: oxygenated hemoglobin; HbR: deoxygenated hemoglobin; PFC: prefrontal cortex.

Of the 21 participants, 12 were further preprocessed to enter the feasibility testing in experiment 1 (Table 1). Within these 12 participants, we had a total 180,580 data cubes; among these cubes, 23,900 (13.24%, 23,900/180,580) were labeled as pain and 156,680 (86.76%, 156,680/180,580) were labeled as no-pain. A total of 2 participants’ data were tested in experiment 2; there were 30,820 data cubes in total, with 2000 (6.49%, 2000/30,820) labeled as right-side pain, 2000 (6.49%, 2000/30,820) labeled as left side pain, and 26,820 (87.02%, 26,820/30,820) labeled as no pain. Figure 3 shows representative averaged HbO and HbR responses across all channels under pain and no-pain statues, respectively, collected from patient 3.

**Table 1:** Participant demographics with classification performance.

Participant	Pain (points)	No-pain (points)	Class accuracy	Reported NRS^a^	Stimulation side
3	2000	14,980	84.58 (%)	5.5	Right
5	2000	13,080	76.83 (%)	3.9	Right
10	2000	12,140	78.84 (%)	5.8	Right
11	2000	11,520	80.98 (%)	1.9	Left
12	2000	13,320	81.53 (%)	3.4	Left
13	2000	13,700	76.25 (%)	8.5	Right
15	2000	11,480	76.12 (%)	5.8	Left
16	2000	12,600	79.55 (%)	6.6	Left
17	1900	14,360	80.74 (%)	2.6	Left
18	2000	13,020	82.29 (%)	3.8	Left
19	2000	11,840	81.00 (%)	5.1	Left
20	2000	14,640	85.78 (%)	3.3	Left

^a^NRS: numerical rating scale.

**Table 2:** Performance of different network setups in experiment 1.

Network setup	Overall accuracy	Sensitivity	Specificity	PPV^a^	NPV^b^	PLR^c^	Kappa
CNN^d^-7	79.62 (%)	0.144	0.896	0.169	0.872	1.39	0.04
CNN-5	79.25 (%)	0.153	0.891	0.183	0.872	1.4	0.05
ANN^e^	79.17 (%)	0.192	0.884	0.205	0.877	1.65	0.08
ANN+2 portion	80.37 (%)	0.326	0.861	0.266	0.893	2.35	0.17
ANN+2 portion + oversample	75.93 (%)	0.409	0.801	0.242	0.898	2.06	0.16
ANN+2 portion + oversample (HbO^f^ only)	77.19 (%)	0.379	0.819	0.245	0.895	2.10	0.16
RNN^g^+2 portion + oversample	76.31 (%)	0.332	0.815	0.211	0.888	1.80	0.11
LSTM^h^+2 portion + oversample	77.29 (%)	0.319	0.828	0.220	0.887	1.86	0.12

^a^PPV: positive predictive value.

^b^NPV: negative predictive value.

^c^PLR: positive likelihood ratio.

^d^CNN: convolutional neural network.

^e^ANN: artificial neural network.

^f^HbO: oxygenated hemoglobin.

^g^RNN: recurrent neural network.

^h^LSTM: long short-term memory.

**Table 3:** Performance of different network setups in experiment 2.

Network	Accuracy	Sensitivity	Specificity	PPV^a^	NPV^b^	PLR^c^	Kappa
ANN^d^	70.88 (%)	0.443	0.777	0.339	0.844	1.99	0.20
CNN^e^-5	65.37 (%)	0.375	0.723	0.250	0.824	1.35	0.08
CNN-6	74.23 (%)	0.279	0.862	0.342	0.823	2.02	0.15
CNN-7	73.23 (%)	0.540	0.782	0.389	0.868	2.48	0.28

^a^PPV: positive predictive value.

^b^NPV: negative predictive value.

^c^PLR: positive likelihood ratio.

^d^ANN: artificial neural network.

^e^CNN: convolutional neural network.

ANN performed on split data history segments achieved the best results, with a prediction accuracy at 80.37 % 145,210/180,580), and a PLR at 2.35 (sensitivity=0.326, specificity=0.861). ANN performed on split data history blocks with reweighted loss function achieved the highest sensitivity at 0.409 and specificity of 0.801, with a PLR at 2.06. In addition, CNN-7 achieved the highest specificity at 0.896, however, with a PLR at 1.39 and a sensitivity of 0.144. A detailed performance summary of experiment 1 for different network setups can be found in Table 2. In addition, a patient-wise classification accuracy was listed in Table 1. The 3-class prediction achieved an optimal classification accuracy at 74.23% (1040/1401) with CNN-6, with a PLR at 2.02 in real time, whereas CNN-7 had a highest sensitivity at 0.540 with a PLR at 2.48. A detailed performance summary of experiment 2 can be found in Table 3.

### Experiment 3

**Figure 4:**
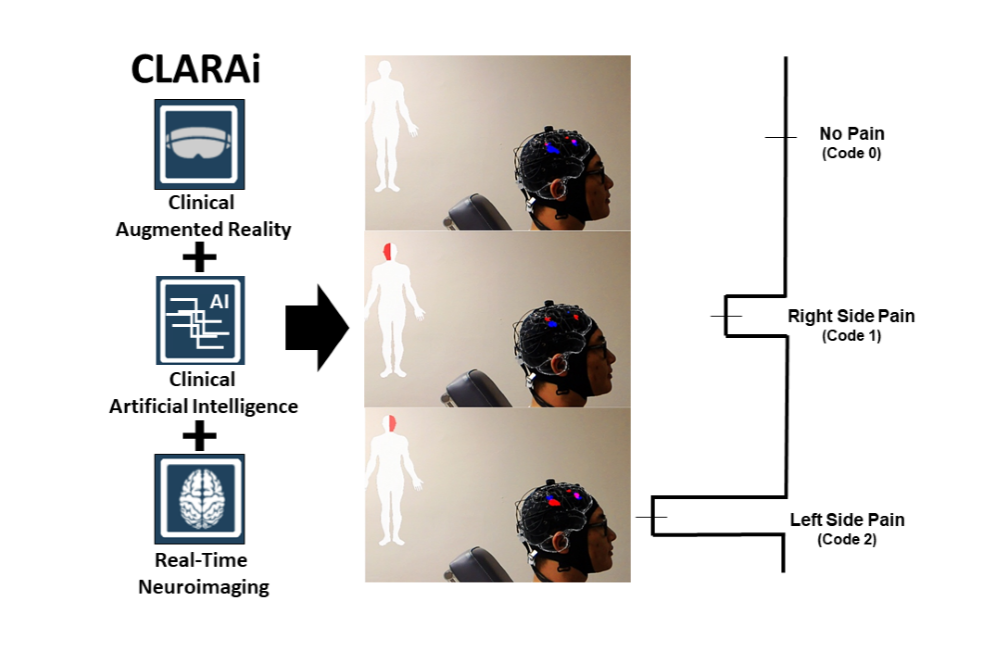
CLARAi framework that integrated clinical real-time neuroimaging, augmented reality, and artificial intelligence provides an augmented clinical environment by displaying neuroimaging data with predicted and localized pain of patient. The classification codes for no-pain, right side pain, and left side pain was defined as 0, 1, and 2, respectively, for model training purposes.

Figure 4 shows the developed data displaying interface “HoloBrain.” The collected functional HbO and HbR data were displayed and updated on an MNI152 brain template in real time (Multimedia Appendix 1; video section 1). The 3D virtual brain activation image, through HoloLens, was superimposed onto a participant’s head. Beside the 3D brain activation, an animated human body with modulated red areas were indicating pain regions prediction by side—either left or right cranio-orofacial regions (Multimedia Appendix 1; video section 2).

## Discussion

In our previous study, we observed, respectively, clinical pain expectation and pain-related responses at PFC and S1 cortices [13]. Further in our successive report, we discovered the sequential connections among those cortical responses, meaning the cascade of cortical events in the brain before, during, and after the clinical pain experience [14]. In this feasibility study, we assembled that pattern for data signature for objective clinical pain prediction using fNIRS data collected directly from the bilateral PFC and S1 cortices. Previous studies achieved promising results in attempting to classify different level of thermal stimulation (potentially indicating pain vs no-pain) based on hemodynamic response data collected from sensory cortex [25,26]. These studies also examined the performance of several prevalent machine learning methods including support vector machine, linear discriminant analysis, and K-nearest neighbor. In our study, we chose to examine the performance of different NN setups, given our data were collected from multiple regions of interest bilaterally including PFCs and S1 cortices at a relatively high sampling rate. On the basis of our trilogy of experiments, our CLARAi model gained not only information from spatial pattern but also temporal sequence in the data by including up to 10-second data history counting back from each data frame to get the contrast between clinical pain expectation and pain experience per se in real time.

Herein, in experiment 1, we tested several NNs on different reorganized brain activation data to predict pain and no-pain conditions. We first tested 3 networks on data including 2-second data history block and found that CNN-7 achieved the highest general classification accuracy. In recent years, CNNs became deeper and deeper, with state-of-the-art networks going from 7 layers AlexNet [27] to a thousand layers Residual Nets [28] in a 4-year period. The reason behind the boost is that a deeper network can usually learn a more complex nonlinear function. Our results on type I data cube complied with such trend that CNN-7 achieved slightly higher accuracy. However, the CNN-7 results emphasized much more specificity than sensitivity, meaning we were likely to better define if the patient had no-pain than pain. Such results may be because of several potential reasons: (1) The collected data were unbalanced, meaning we had much more no-pain than pain samples in the training set from each patient; (2) We did not have enough data to train the large number of parameters for CNN; and (3) The pattern contained within the 2-second data history may not be informative enough for discriminating pain from no-pain condition. Therefore, to improve sensitivity, we employed a simpler ANN network, reweighted the loss function relevant to the pain condition samples, and assembled data feature with 2 split history blocks to include pain expectation evoked responses [29]. When the preceding pain expectation (prepain) phase is also taken into consideration in the algorithm [29], the results indicated that the real time sensitivity was significantly increased to detect pain, from 0.144 to 0.409, while not losing too much specificity, from 0.896 to 0.861. Such improvement in pain detection (sensitivity) suggested that the pain expectation phase was crucial to encode the magnitude of the immediate pain and is highly driven by the activation of the left dorsolateral PFC [14,30]. In addition, we tested the same NN setup on only HbO data. We found a slight increase in general classification accuracy and specificity (0.013 and 0.018), whereas a decrease in sensitivity (0.029). As sensitivity was generally lower than specificity in this study, we selected combined HbO and HbR data as features for classification. Finally, we examined whether there was a potential correlation between the individual level classification accuracy and the reported numerical rating scale of pain, but we did not find any statistical significance.

Considering the relatively high-spatial resolution of fNIRS imaging, in experiment 2, we further tested the feasibility of localizing pain. We introduced a 3-class pain localization problem by merging the data from 2 selected patients, one with left side tooth pain hypersensitivity and the other one with same clinical condition on a right tooth during cold stimulation. To eliminate baseline and signal magnitude difference, we applied a simulated real-time normalization algorithm to the data. We then tested this dataset with several NNs with different depth and found that CNN-6 achieved the best general classification accuracy. Though there is need for further validation, the preliminary discrimination result demonstrated a strong potential of our framework in localizing pain at different body regions. Moreover, the results demonstrated the feasibility of training a universal model that can localize pain condition across patients based on the S1 homuncular activation by side and major body regions. This is biologically feasible because the somatotopic homuncular S1 representation for pain in the orofacial region is quite large, like other major functional body regions including the thumb/hand, trunk, and feet [31].

Combined with the pain prediction module, we developed a clinical AR-based data displaying interface for the framework. The data collected in this study with the predicted result were transferred to a HoloLens device. The magnitudes of hemodynamic response changes at multiple locations on a 3D brain template were superimposed on the participants’ head in reality via HoloLens (Multimedia Appendix 1; video 1). The predicted painful locations were indicated by the red flickering parts on the animated body beside the virtual brain. In a true clinical environment, with such framework, clinicians can better understand in an objective way to determine when/where the patients are suffering from pain, especially when they cannot express themselves. In addition, the potential idea is to even decide the level of pain, and further a “prepain” phase using PFC activation evoked by pain-associated anxiety or expectation. Such information will help clinicians decide when to intervene for addressing the pain or the immediate likelihood of it to occur (eg, anesthesia and brain stimulation). Afterwards, the entire framework becomes closed loop by including a pain intervention module, for instance pain neuromodulation.

Finally, all selected data preprocessing, classification, transmitting, and displaying methods in this study can be implemented in real time. However, the CLARAi framework is in its initial stages. Future improvements of this work include: (1) optimizing the framework sensitivity by potentially adding short-separation channels during data acquisition to model interfering physiological signals in a better way, (2) expansion of the current participant-specific model to a general model with learning ability that will only require individualization to precisely adapt to variations in each patient, and (3) further expansion of the model to fit other types of pain conditions and neurologic disorders including depression and anxiety. In summary, we tested the feasibility of a prototype of a mobile neuroimaging-based clinical AR and AI (CLARAi) framework for objective pain detection and localization in the clinical environment in real time. Such framework predicted when and where there was physical pain based on the brain statuses in our data study and displayed neuroimaging data interactively in real time. Although extensive validation work still needs to be done, the CLARAi framework might turn into reality the goal of precisely “seeing and believing” the biologic pain suffering of our patients in the doctor’s office.

## Appendix

Multimedia Appendix 1 Video: section 1, the collected oxygenated hemoglobin and deoxygenated hemoglobin data were displayed on an MNI152 brain template in real time. Section 2, the 3-dimensional (3D) virtual brain activation image, through HoloLens, was superimposed onto a participant’s head. Beside the 3D brain activation, an animated human body with modulating red areas indicated pain regions prediction by side—either left or right cranio-orofacial regions.

## Abbreviations

3D: 3-dimensional
AI: artificial intelligence
ANN: artificial neural network
AR: augmented reality
CNN: convolutional neural network
fMRI: functional magnetic resonance imaging
fNIRS: functional near-infrared spectroscopy
HbO: oxygenated hemoglobin
HbR: deoxygenated hemoglobin
NN: neural network
PLR: positive likelihood
S1: primary sensory
